# Targeting the *Wolbachia* Cell Division Protein FtsZ as a New Approach for Antifilarial Therapy

**DOI:** 10.1371/journal.pntd.0001411

**Published:** 2011-11-29

**Authors:** Zhiru Li, Amanda L. Garner, Christian Gloeckner, Kim D. Janda, Clotilde K. Carlow

**Affiliations:** 1 New England Biolabs, Division of Parasitology, Ipswich, Massachusetts, United States of America; 2 Departments of Chemistry and Immunology and Microbial Science, The Skaggs Institute for Chemical Biology, and The Worm Institute for Research and Medicine, The Scripps Research Institute, La Jolla, California, United States of America; New York Blood Center, United States of America

## Abstract

The use of antibiotics targeting the obligate bacterial endosymbiont *Wolbachia* of filarial parasites has been validated as an approach for controlling filarial infection in animals and humans. Availability of genomic sequences for the *Wolbachia* (*w*Bm) present in the human filarial parasite *Brugia malayi* has enabled genome-wide searching for new potential drug targets. In the present study, we investigated the cell division machinery of *w*Bm and determined that it possesses the essential cell division gene *ftsZ* which was expressed in all developmental stages of *B. malayi* examined. FtsZ is a GTPase thereby making the protein an attractive *Wolbachia* drug target. We described the molecular characterization and catalytic properties of *Wolbachia* FtsZ. We also demonstrated that the GTPase activity was inhibited by the natural product, berberine, and small molecule inhibitors identified from a high-throughput screen. Furthermore, berberine was also effective in reducing motility and reproduction in *B. malayi* parasites *in vitro*. Our results should facilitate the discovery of selective inhibitors of FtsZ as a novel anti-symbiotic approach for controlling filarial infection.

**Note:**

The nucleotide sequences reported in this paper are available in GenBank™ Data Bank under the accession number *wAlB*-FtsZ (JN616286).

## Author Summary

Filarial nematode parasites are responsible for a number of devastating diseases in humans and animals. These include lymphatic filariasis and onchocerciasis that afflict 150 million people in the tropics and threaten the health of over one billion. The parasites possess intracellular bacteria, *Wolbachia*, which are needed for worm survival. Clearance of these bacteria with certain antibiotics leads to parasite death. These findings have pioneered the approach of using antibiotics to treat and control filarial infections. In the present study, we have investigated the cell division process in *Wolbachia* for new drug target discovery. We have identified the essential cell division protein FtsZ, which has a GTPase activity, as an attractive *Wolbachia* drug target. We describe the molecular characterization and catalytic properties of the enzyme and demonstrate that the GTPase activity is inhibited by the natural product, berberine, and small molecule inhibitors identified from a high-throughput screen. We also found that berberine was effective in reducing motility and reproduction in *B. malayi* parasites *in vitro*. Our results should facilitate the discovery of selective inhibitors of FtsZ as a novel antibiotic approach for controlling filarial infection.

## Introduction

Filarial nematode parasites are responsible for a number of devastating diseases in humans and animals. These include lymphatic filariasis and onchocerciasis that afflict 150 million people in the tropics and threaten the health of over one billion. Unlike other nematodes, the majority of filarial species are infected with an intracellular bacterium, *Wolbachia* [1]. In the human filarial nematode *Brugia malayi*, these obligate α-proteobacterial endosymbionts have been detected in all developmental stages [2]–[4]. Moreover, their presence is essential for the worm, as tetracycline-mediated clearance of bacteria from *Brugia* spp. leads to developmental arrest in immature stages and reduction in adult worm fertility and viability [5]–[10]. These findings have pioneered the approach of using antibiotics to treat and control filarial infections. However, in humans, tetracycline therapy is not ideally suited for widespread use because several weeks of treatment are required and the drug has contra-indications for certain individuals. Therefore, there is considerable interest in identifying new endosymbiont drug targets and other classes of compounds with anti-*Wolbachia* activity. Importantly, the completed genome sequence of the *Wolbachia* endosymbiont of *B. malayi* (*w*Bm) [11] now enables genome-wide mining for new drug targets [11]–[14] and a foundation for rational drug design. These approaches should lead to the discovery of new classes of compounds with potent anti-*Wolbachia*/antifilarial activities targeting essential processes that are absent or substantially different in the mammalian host.

Bacterial cytokinesis has emerged as a major target for the design of novel antibacterial drugs [15]–[17] since several of the components that are essential for multiplication and viability are absent from mammals. The bacteria-specific “filamenting temperature sensitive” protein, FtsZ, plays a central role during bacterial cytokinesis. In *Escherichia coli*, temperature sensitive mutations in the *ftsZ* gene cause blockage in cell division with limited cell growth and the generation of long filaments. FtsZ assembles into the contractile Z-ring and coordinates more than a dozen other cell division proteins at the midcell site of the closing septum [18]–[21]. Formation of the septal Z-ring requires two important functional properties of FtsZ, namely, polymerization of the FtsZ monomers into protofilaments and GTPase activity. Since inhibition of either function is lethal to bacteria, both GTP-dependent polymerization [22]–[27] and enzymatic [27]–[28] activities of FtsZ have been targeted for the identification of new antibacterial agents. Several inhibitors have been discovered including synthetic compounds [17], [29] and natural products [17], [30]–[33].

In the present study, we identify the cell division machinery present in *w*Bm and characterize the FtsZ protein (*w*Bm-FtsZ). Using quantitative real time RT-PCR, *Wolbachia ftsZ* was found to be expressed throughout the life cycle, but up-regulated in fourth stage larvae and adult female worms. Recombinant *w*Bm-FtsZ was shown to possess a robust GTPase activity, which was inhibited by the natural plant product berberine. Berberine was also effective in reducing motility and reproduction in *B. malayi* parasites *in vitro*. A library of small molecules was also examined for its inhibitory activity against the *w*Bm and *E. coli* FtsZ proteins. Several compounds were identified as potent inhibitors, and structure-activity relationship studies revealed a derivative with selectivity for *w*Bm-FtsZ. Thus, our results support the development of *w*Bm-FtsZ as a promising new drug target in an anti-symbiotic approach for controlling filarial infection.

## Materials and Methods

### Cloning of *ftsZ* from the *Wolbachia* endosymbiont of the human filarial parasite *B. malayi* (*w*Bm-*ftsZ*)

Living *B. malayi* adult female worms were purchased from TRS Laboratories, Athens GA. Genomic DNA and RNA were isolated following the protocols developed by Dr. Steven A. Williams (http://www.filariasiscenter.org/molecular-resources/protocols).

To clone full-length *wBm-ftsZ* for expression studies, forward 5′(GAGAGCTAGCATGTCAATTGACCTTAGTTTGCCAG)3′ (NheI site underlined) and reverse 5′(GAGACTCGAGTTACTTCTTTCTTCTTAAATAAGCTGG) 3′ (XhoI site underlined) primers were designed according to the *wBm*-*ftsZ* sequence (accession number: YP_198432) in order to amplify the gene from *B. malayi* genomic DNA. The PCR product was then cloned into the NheI and XhoI sites of pET28a(+) (Novagen) to generate a fusion protein with a His_6_ tag at the N terminus. The authenticity of the insert was verified by sequencing.

### *Wolbachia ftsZ* gene expression in various developmental stages of *B. malayi*

Total RNA supplied by the Filariasis Research Resource Center (FR3) was treated with RNase-free Dnase (New England Biolabs, Cat# M0303S) and purified using the RNeasy Kit from Qiagen. cDNA was obtained using random primers and the ProtoScript® AMV First Strand cDNA Synthesis Kit (New England Biolabs, Cat# E6550S). Forward primer 5′ (AACAAGAGAGGCAAGAGCTGGAGT) and reverse primer 5′(CGCACACCTTCAAAGCCAAATGGT) were utilized to amplify a 102 bp *Wolbachia ftsZ* amplicon. *Wolbachia* 16S rRNA amplified with forward primer 5′ (TGAGATGTTGGGTTAAGTCCCGCA) and reverse primer 5′(ATTGTAGCACGTGTGTAGCCCACT) was utilized for bacterial total RNA quantification. *B. malayi* 18S rRNA amplified with forward primer 5′ (ACTGGAGGAATCAGCGTGCTGTAA) and reverse primer 5′(TGTGTACAAAGGGCAGGGACGTAA) was utilized as a total worm RNA control. Quantitative PCR was performed using the DyNAmo™ HS SYBR® Green qPCR Kit (Thermo Fisher) and a *CFX-96* Real Time PCR instrument (Bio-rad, Hercules, CA). Relative levels of *ftsZ* expression (ratio of *ftsZ* to 16S rRNA), and abundance of *Wolbachia* in *B. malayi* (ratio of *Wolbachia* 16S to *B. malayi* 18S rRNA) were calculated for each RNA sample. Experiments were performed twice with triplicate samples. Controls consisting of samples processed in the absence of reverse transcriptase were included in qPCR and no DNA contamination was detected.

### Identification and cloning of FtsZ from the *Wolbachia* endosymbiont of *Aedes albopictus*

To determine the sequence of the *ftsZ* gene from the *Wolbachia* endosymbiont *wAlB* present in the insect cell line Aa23 [34], multilocus sequence typing (MLST) *ftsZ* forward 5′ (TGTAAAACGACGGCCAGTATYATGGARCATATAAARGATAG) and reverse 5′ (CAGGAAACAGCTATGACCTCRAGYAATGGATTRGATAT) [35] primers were utilized to obtain a PCR fragment. Using BLAST analysis, the sequence of the PCR product was compared to the corresponding region of known full-length *ftsZ* sequences and their conserved downstream and upstream sequences and 6 additional primers 5′(TCTATTTTTAATTCTTTTAGAGAAGCATT), 5′(CGTTCGGTTTTGAAGGTGTGC), 5′ (ACCGTTGTGGGAGTGGGTGGT), 5′ (TTATTTTTTTCTTCTTAAATAAGCTGGTATATC), 5′ (GGAATGACAATAAGTGTATCTACGTA), and 5′(TGCATTTGCAGTTGCTCATCC) were designed to obtain a complete *wAa*-*ftsZ* sequence. Phusion® High-Fidelity DNA Polymerase (New England Biolabs, M0530) was utilized for all PCR reactions according to manufacturer's instructions.

### Expression and purification of recombinant *Wolbachia* FtsZ proteins

*w*Bm-*ftsZ* and *E. coli ftsZ (Ec-ftsZ)* were amplified using genomic DNA isolated from *B. malayi* and *E. coli wild-type strain* MG1655 respectively, and were then cloned into the pET28a plasmid to generate fusion proteins with a N-terminal His tag. Each protein was expressed in the *Escherichia coli* strain C2566 (New England Biolabs). Optimum conditions for production of soluble recombinant *w*Bm-FtsZ involved co-transformation with the pRIL plasmid isolated from BL21-CodonPlus (DE3) cells (Stratagene) together with the pET28a-ftsZ plasmid. Cultures were grown at 37°C till the OD_600_ reached 0.6, before induction with 0.1 mM IPTG overnight at 16°C. Both Ec-FtsZ and *w*Bm-FtsZ were purified using a similar method. The cells expressing the recombinant proteins were suspended in lysis buffer (20 mM NaPO_4_, 500 mM NaCl, 10 mM imidazole, pH 7.4) plus 1 mg/mL lysozyme and protease inhibitor cocktail (Roche) and incubated on ice for 30 min, followed by sonication. The lysate was then cleared by centrifugation at 12,500 rpm, 4 °C for 30 min. The His-tagged proteins were purified on a 5 mL HiTrap chelating HP column (GE Healthcare) using an AKTA FPLC following manufacturer's instructions. After application of the sample, the column was washed with 5 column volumes of buffer A (20 mM NaPO_4_, 500 mM NaCl, 10 mM imidazole, pH 7.4) followed by 10 column volumes of 92% buffer A:8% buffer B (20 mM NaPO_4_, 500 mM NaCl, 400 mM imidazole, pH 7.4). Protein was then eluted using a linear gradient (8–100%) of buffer B equivalent to 40–400 mM imidazole.

Fractions containing *w*Bm-FtsZ or Ec-FtsZ were pooled, dialyzed against dialysis buffer (40 mM Tris-HCl, 200 mM NaCl and 50% glycerol, pH 7.5) and stored at −20°C prior to use. Purity of the proteins was estimated by 4–20% SDS-PAGE and the protein concentration was determined using the Bradford assay.

### GTPase enzyme assay

GTPase activity was measured using an enzyme-coupled assay [36]. Activity was determined by measuring the consumption of NADH, which is monitored by absorbance at 340 nm. The amount of NADH oxidized to NAD corresponds to the amount of GDP produced in the reaction. Reactions were optimized for a 96-well format to enable compound screening. The 100 µL reaction mixture containing 50 mM MOPS (4-morpholinepropanesulfonic acid) pH 6.5, 50 mM KCl, 5 mM MgCl2,1 mM PEP, 500 mM NADH, 0.1% Tween-20, 20 units/mL of L-lactate dehydrogenase (Sigma L2518) and pyruvate kinase (Sigma P7768), 1 mM GTP and 5 mM FtsZ was distributed into 96-well plates. The plate was incubated at 30 °C for 45 min with data collected at 20 second intervals using a SpectraMax® Plus 384 (Molecular Devices) spectrophotometer. Control assays without FtsZ were performed to provide a baseline and with GDP to ensure the function of the coupling enzymes.

For inhibitor screening, 100 µL of reaction mixture was added to each well of a 96-well plate and 1 µL of compound dissolved in DMSO, or berberine sulfate (MP Biomedicals) in water, in varying concentrations were added. The reaction was initiated at 30 °C by adding 1 mM GTP. Experiments were performed in triplicate.

### Effect of berberine on *B. malayi*

Living *B. malayi* adult female and male worms were washed extensively with RPMI1640 medium supplemented with 2 mM glutamine, 10% Fetal Calf Serum (Gibco) and 100 U/mL streptomycin, 100 mg/mL penicillin, 0.25 mg/mL amphotericin B (Sigma). Three worms of either gender were distributed into each well of a 6-well plate and incubated at 37 °C, 5% CO_2_. After overnight recovery, motility and microfilaria production were recorded. Worms were then transferred to a new well containing varying amounts of berberine sulfate dissolved in water, namely 40 µM, 20 µM, 10 µM and 5 µM. Control wells containing either no drug or 10 µM doxycycline, were also included. Culture media were replaced with fresh medium containing drug daily. Adult worm and microfilaria motility production were recorded daily as described [37]. Motility was scored as described [38] and expressed as % of motility relative to motility scored on day 0 of the experiment. Microfilaria production was counted in 10 µL of either diluted or concentrated culture medium using a hemocytometer. The results were presented as the number of microfilaria released in 1 mL of medium from each well on the indicated day. Each treatment was performed in triplicate and the experiment was repeated several times.

### Effect of berberine on *E. coli* growth

Berberine sulfate (MP Biomedicals) was added at a final concentration of 0–400 µM to growth medium containing *E. coli* ER1613 (*acrA13 Δ(top-cysB)204 gyrB225 IN(rmD-rmE) mcrA*) (New England Biolabs) and growth determined during 5 h or 20 h of incubation. For the 5 h evaluation, an overnight culture of *E. coli* ER1613 (*acrA13 Δ(top-cysB)204 gyrB225 IN(rmD-rmE) mcrA*) (New England Biolabs) was diluted 100-fold and 1 mL volumes were dispensed into a 48-well deep well plate (Axygen Scientific) containing various concentrations (0–400 µM) of berberine sulfate (10 µL of serial diluted berberine sulfate in water). The plate was then incubated at 30 °C with shaking. After 90 min of initial growth, bacterial growth was determined every 30 min for 5 h by monitoring absorption at 600 nm using a microtiter plate reader (Spectramax M5, Molecular Devices). Alternatively, an overnight culture of *E. coli* was diluted 1∶1000 fold and incubated with varying amounts of berberine sulfate for 20 h before growth was determined. All experiments were performed at least twice. Viability of berberine sulfate-treated (24 h) cells was evaluated by spotting 3 µL serial dilutions (10^−2^–10^−7^) of bacteria on a petri dish and incubation overnight at 30 °C.

Bacterial morphology was visualized using a Zeiss AxioVert 200 microscope and images were obtained using a 20× objective.

### General chemistry methods for library synthesis

Reactions were carried out under a nitrogen atmosphere with dry, freshly distilled solvents under anhydrous conditions, unless otherwise noted. Yields refer to chromatographically and spectroscopically homogenous materials, unless otherwise stated. Reactions were monitored by thin-layer chromatography (TLC) carried out on 0.25-mm EMD silica gel plates (60F-254) using UV-light (254 nm). Flash chromatography separations were performed on Silicycle silica gel (40–63 mesh). Purity analyses were performed using HPLC (254 nm).

### General synthetic procedure for library compounds

A stirring solution of aldehyde (1.0 equiv) in MeOH at 25°C was treated with carboxylic acid (2.0 equiv), amine (2.0 equiv) and isonitrile (2.0 equiv). The solution was heated to reflux, and stirred for 24 h. The solution was then cooled to 25°C and concentrated *in vacuo*. The crude residue was purified via flash column chromatography (10–50% EtOAc in hexanes) to afford the purified product. For characterization data, see references [39]–[40].

## Results

### Genomic organization of the major cell division genes in *w*Bm

The bacterial cell-division pathway has been extensively studied in *E. coli* and several essential proteins have been identified [17], [19]. Many of the genes encoding putative orthologs of these proteins are also present in *w*Bm (Table 1). A total of 18 major cell division genes were identified in *w*Bm genome (Table 1), including *ftsZ*, *ftsA*, *ftsI*, *ftsK*, *ftsQ* and *ftsW*, which are known to be essential for cell division [17]. These *w*Bm genes were mapped and found to be more scattered throughout the genome, in comparison with their *E. coli* homologs. In *E. coli* the majority of genes were found in one major operon, with the remaining 5 genes distributed randomly. Of these, FtsZ was one of the most highly conserved essential proteins possessing 43% identity to *Ec*-FtsZ (Table 1). *Wolbachia ftsA*, *ftsI*, *ftsK*, *ftsQ* and *ftsW* were less related (13–34%) to the *E. coli* homologs. Some previously described essential cell division genes in *E. coli* (including *ftsB*, *ftsL*, *ftsN* and *ZipA*) were not found in *w*Bm, indicating that there are differences in the cell division machinery present in free living *E. coli* and intracellular *Wolbachia*.

**Table 1:** Comparison of cell division machinery present in *Wolbachia* and *E. coli* *.

Gene name	Ec number	Ec Size (AA)	wbm gene number	wBm Size (AA)	Annotation	Identity
*ftsW*	b0089	414	wbm0015	373	integral membrane protein involved in stabilizing FstZ ring during cell division	24.4
*zapA*	b2910	109	wbm0057	105	protein that localizes to the cytokinetic ring	18.1
*ftsI*	b0084	588	wbm0075	521	transpeptidase in septal peptidoglycan synthesis (penicillin-binding protein 3)	18.6
*mraW*	b0082	313	wbm0107	333	16S rRNA m(4)C1402 methyltranserfase, SAM-dependent	34.2
*ftsA*	b0094	420	wbm0113	412	ATP-binding cell division protein involved in recruitment of FtsK to Z ring	23.3
*murC*	b0091	491	wbm0118	556	UDP-N-acetylmuramate:L-alanine ligase	27.7
*mreB*	b3251	347	wbm0154	358	cell wall structural complex MreBCD, actin-like component MreB	53.0
*murF*	b0086	452	wbm0238	455	UDP-N-acetylmuramoyl-tripeptide:D-alanyl-D-alanine ligase	27.9
*ftsH*	b3178	644	wbm0490	609	protease, ATP-dependent zinc-metallo	46.5
*murE*	b0085	495	wbm0492	496	UDP-N-acetylmuramoyl-L-alanyl-D-glutamate:meso-diaminopimelate ligase	25.2
*murD*	b0088	438	wbm0508	498	UDP-N-acetylmuramoyl-L-alanine:D-glutamate ligase	22.8
*murG*	b0090	355	wbm0557	343	N-acetylglucosaminyl transferase	22.4
*ddl*	b0092	306	wbm0570	339	D-alanine:D-alanine ligase	29.4
*ftsQ*	b0093	276	wbm0571	252	Divisome assembly protein, membrane anchored protein at septum	13.1
*ftsZ*	b0095	383	wbm0602	396	GTP-binding tubulin-like cell division protein	42.8
*mraY*	b0087	360	wbm0643	326	phospho-N-acetylmuramoyl-pentapeptide transferase	33.1
*ftsK*	b0890	1329	wbm0644	707	DNA translocase at septal ring sorting daughter chromsomes	33.7
*murB*	b3972	342	wbm0778	295	UDP-N-acetylenolpyruvoylglucosamine reductase, FAD-binding	19.3

*Only major *E. coli* cell-division proteins are shown.

### Sequence analysis of *w*Bm-*ftsZ*

*w*Bm-*ftsZ* exists as a single gene on the chromosome and is 1182 bp in length. It encodes a 394-amino acid protein with a predicted molecular mass of 42 kDa containing four distinct domains characteristic of FtsZ proteins. These comprise the variable N-terminal domain, a highly conserved core region, variable spacer, and a C-terminal conserved domain. The core region contains the highly conserved catalytic aspartate residue [41]–[42] and the GGGTGTGA motif (8 residues see [41], [43]), which are responsible for GTP hydrolysis and required for polymerization of the protein. The C-terminal region is not required for assembly, but is essential for interactions with the cell division proteins FtsA, FtsW and ZipA [17]. A similar organization was also found in the insect Wolbachia, *wMel*-FtsZ (NP_966481) and *wAlB*-FtsZ (JN616286). The FtsZ proteins of *Wolbachia* from different hosts share 89–91% identity and 43% identity to *E. coli FtsZ* proteins, with a substantially lower level at the carboxyl-terminal region (17.2% identity).

### Analysis of *w*Bm-*ftsZ* expression during the life cycle of *B. malayi*

*Wolbachia* have been identified in all developmental stages of *B. malayi*, from studies on individual worms and isolates from regions endemic for lymphatic filariasis [2]–[4]. To determine the relative expression of *w*Bm-FtsZ throughout the parasite life cycle and validate its suitability as a drug target, *w*Bm-*ftsZ* mRNA expression was analyzed by quantitative real-time reverse transcription polymerase chain reaction (qRT-PCR). Relative levels of *ftsZ* expression (ratio of *Wolbachia ftsZ* to 16S rRNA) and abundance of *Wolbachia* in *B. malayi* (ratio of *Wolbachia* 16S to *B. malayi* 18S rRNA) were calculated for each RNA sample.

**Figure 1:**
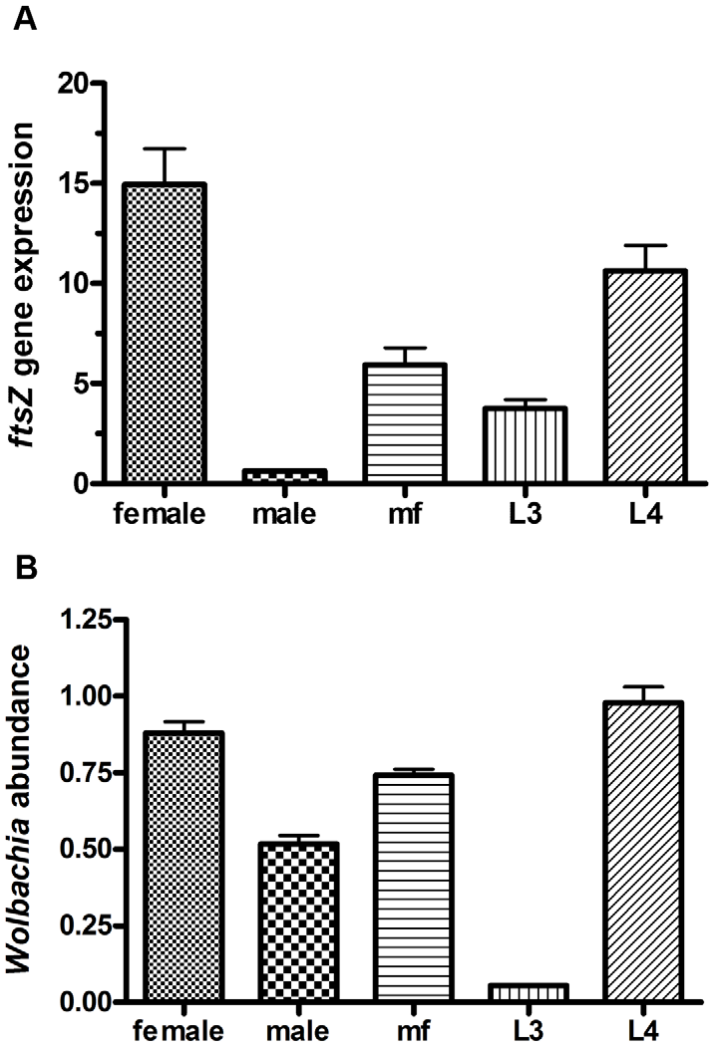
*Wolbachia ftsZ* gene expression in various developmental stages of *B. malayi*. Female adult worm, male adult worm, microfilaria, L3 and L4 were analyzed. The ratio of ftsZ to 16S rRNA (A) represents *ftsZ* gene expression, while the ratio of *Wolbachia* 16S to *B. malayi* 18S rRNA (B) represents the relative abundance of *Wolbachia* in *B. malayi*. The data obtained from triplicate samples are expressed as a mean ± standard deviation.

*w*Bm-*ftsZ* was found to be expressed throughout all stages examined (adult female and male worms, microfilariae, third- and fourth-stage larvae). Moreover, *w*Bm-*ftsZ*/16S ratios were found to be increased substantially following infection of the mammalian host since levels were significantly higher (p value<0.001) in fourth-stage larvae and adult female worms compared to the vector-derived infective third-stage larvae. The *w*Bm-*ftsZ*/16S ratio was also higher in microfilariae compared with the vector-derived third-stage larvae, but was significantly lower than the ratios obtained for fourth-stage and adult female worms. Of the various developmental stages examined, the lowest level of *w*Bm-*ftsZ* expression was found in male worms (Figure 1A). No DNA contamination was detected in controls consisting of samples processed in the absence of reverse transcriptase. *Wolbachia* 16S rRNA/*B. malayi* 18S rRNA ratios were also determined to measure the relative abundance of bacteria in different stages of *B. malayi* (Figure 1B). *Wolbachia* was found to be most abundant in fourth stage larvae and adult female worms and least abundant in infective third stage larvae, indicating a massive multiplication of *Wolbachia* soon after infection of the mammalian host. Taken together, these data indicate that while *w*Bm-*ftsZ* is expressed in all stages, gene activity and bacterial multiplication is most pronounced in fourth-stage larvae and adult females.

### Expression and purification of recombinant *w*Bm-FtsZ

**Figure 2:**
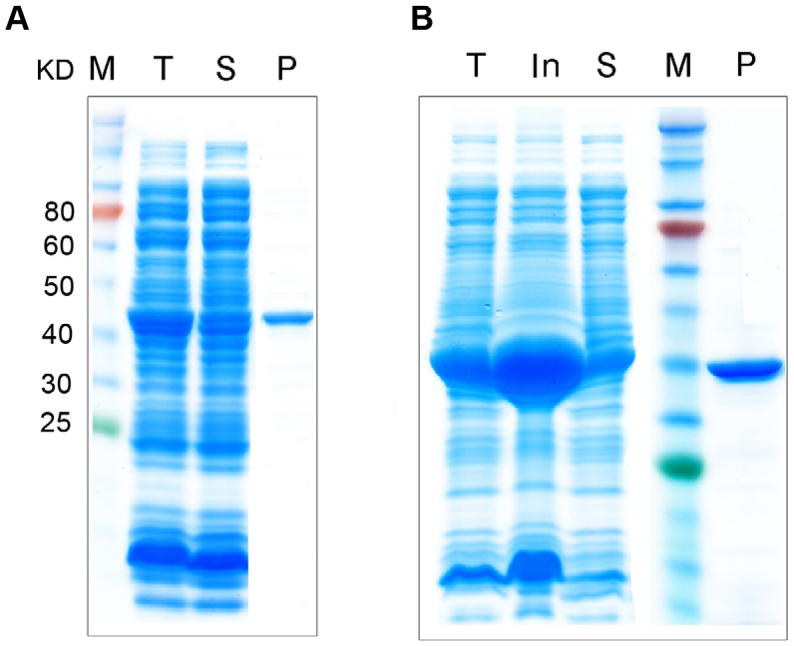
Expression and purification of recombinant FtsZ proteins. FtsZ protein from *Wolbachia* (A) and *E. coli* (B) were expressed in *E. coli* with a His-tag at the N-terminus and purified by nickel-affinity chromatography. The apparent molecular weight (indicated) from SDS–PAGE was consistent with the predicted molecular size. Protein marker (M), total protein lysate (T), insoluble proteins (In), soluble (S) and purified FtsZ proteins (P) are shown.

Recombinant *w*Bm-FtsZ was expressed in *E. coli* with a His-tag at the C-terminus and purified by nickel-affinity chromatography (Figure 2A). Optimum conditions for production of soluble recombinant *w*Bm-FtsZ involved growth of cultures at 37°C until the OD_600_ reached 0.6, followed by induction with 0.1 mM IPTG overnight at 16°C. Purified protein was eluted with 100 mM imidazole. The apparent molecular weight of 43 kDa (Figure 2A) was consistent with the predicted molecular size of *w*Bm-FtsZ with an N-terminal His-tag. For comparative studies, *E. coli* FtsZ (41 kDa) was also expressed and purified in a similar manner (Figure 2B).

### Recombinant *w*Bm-FtsZ has GTPase activity

**Figure 3:**
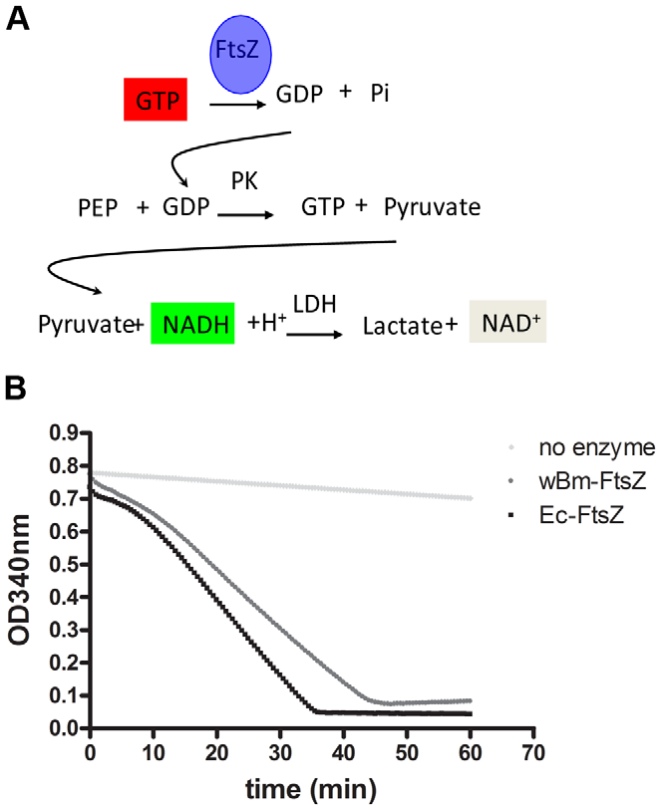
GTPase activity of recombinant *w*Bm-FtsZ. Panel A, activity was determined indirectly by measuring a decrease in NADH concentration by its absorbance at 340 nm. FtsZ hydrolyzes GTP into GDP and inorganic phosphate. The GDP product is used as a substrate by pyruvate kinase (PK) in the presence of phosphoenol pyruvate (PEP) to yield GTP and pyruvic acid as products. Pyruvic acid is used as a substrate in the presence of NADH by lactate dehydrogenase (LDH) to generate lactate and NAD. The consumption of NADH is proportional to GTPase activity. Panel B, comparison of the GTPase activity of *w*Bm-FtsZ and Ec-FtsZ. A control without enzyme was also included. Activity was indicated by a decrease of NADH measured by absorbance at 340 nm.

GTPase activity was measured using an enzyme-coupled assay involving pyruvate kinase and lactate dehydrogenase [36]. GTP hydrolysis was determined by measuring the decrease in fluorescence emission following oxidation of nicotinamide adenine dinucleotide (NADH) to NAD (Figure 3A). As Figure 3B shows, recombinant *w*Bm-FtsZ was found to possess GTPase activity. Moreover, the specific activities for *w*Bm-FtsZ and Ec-FtsZ were comparable (0.18±0.012 µmolµmin^−1^mg^−1^ and 0.22±0.015 µmol min^−1^mg^−1^, respectively).

### Inhibition of *w*Bm-FtsZ GTPase activity using the plant alkaloid berberine

**Figure 4:**
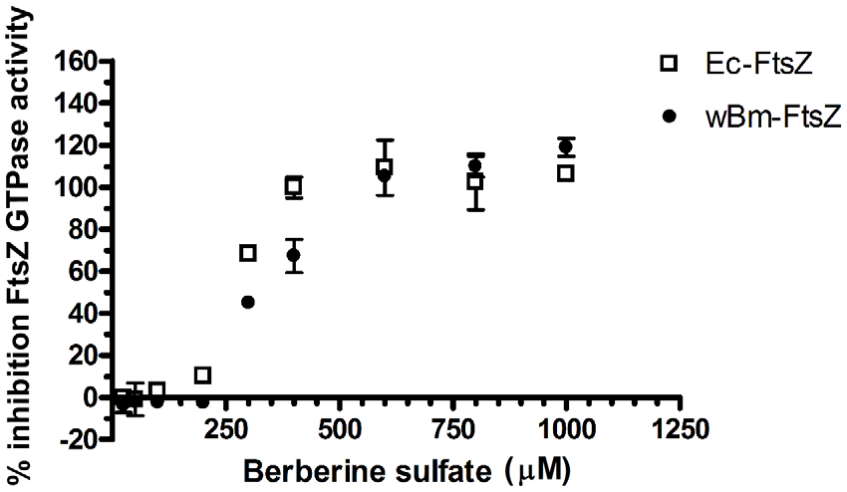
Berberine sulfate inhibition of the GTPase activity. Enzyme activity of *w*Bm-FtsZ (•) and Ec-FtsZ (□) was determined in the presence of 0, 25, 50, 100, 200, 400, 600, 800, and 1000 µM berberine sulfate. The data obtained from triplicate samples are expressed as a mean ± standard deviation.

Berberine, an alkaloid natural product, is a known inhibitor of the GTPase activity of FtsZ in *E. coli* [33], [44]. Thus, we were interested in examining the generality of berberine's GTPase inhibitory activity against *w*Bm-FtsZ. As Figure 4 shows, dose-dependent inhibition (25–1000 µM) was found with an IC_50_ value of 320 µM. *E. coli* FtsZ [33], [44] was included for comparison, and an IC_50_ value of 240 µM was observed (Figure 4). Since *w*Bm-FtsZ possesses all but one of the key residues proposed in the binding of *E. coli* FtsZ to berberine (lysine instead of glycine at position 183 of *Ec*-FtsZ), this may account for the higher concentration of berberine required to inhibit 50% of *w*Bm-Ftsz's GTPase activity.

### The effect of berberine on the motility and microfilariae production of *B. malayi in vitro*

**Figure 5:**
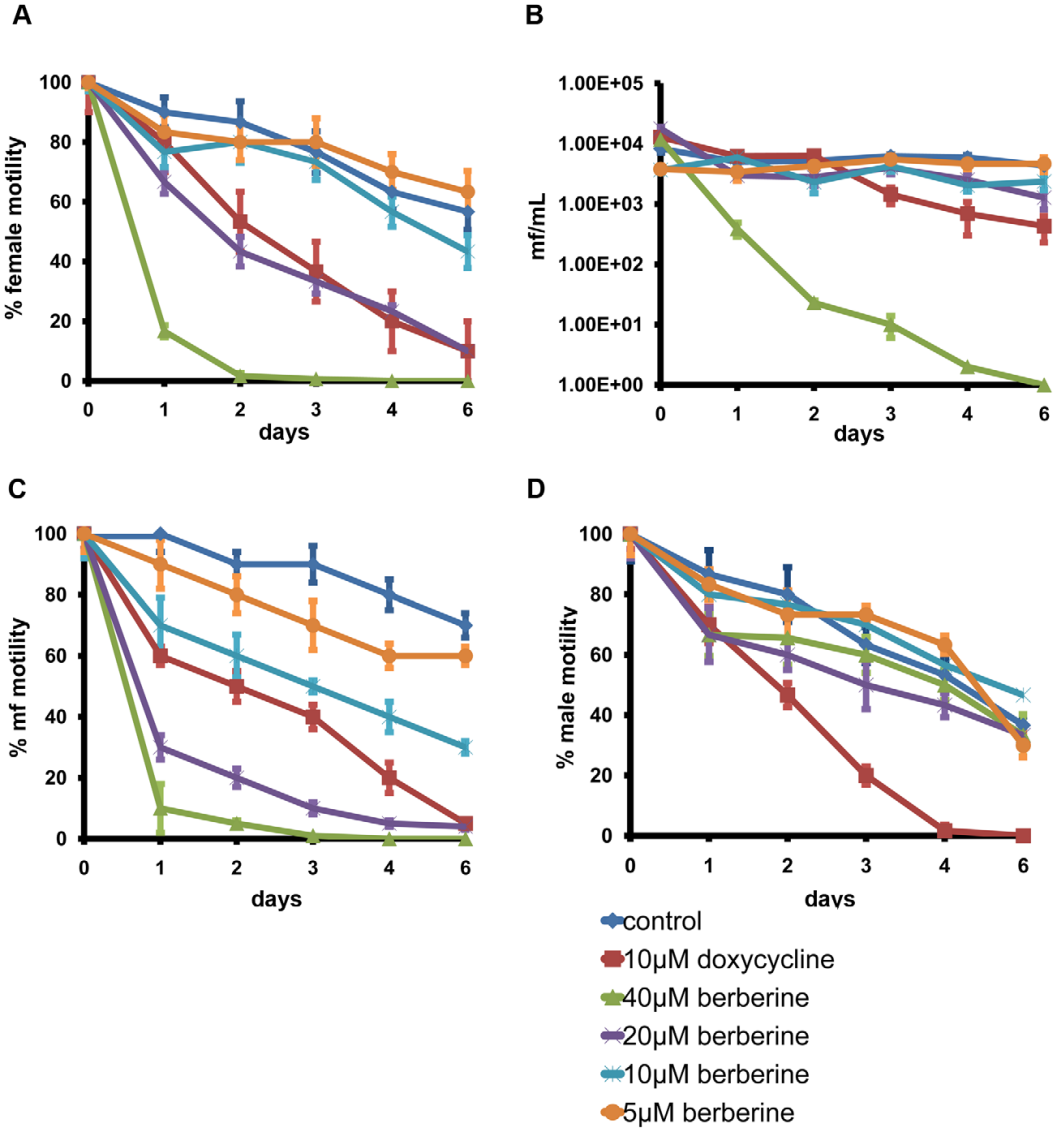
Effect of berberine sulfate on *B. malayi* parasites in culture. Motility of adult female (A) and male (D) worms, and microfilariae (C) was examined following 6 days exposure to varying amounts (5–40 µM) of berberine sulfate. 10 µM doxycycline was included as a control. Motility was scored as described [38] and expressed as % of motility relative to motility scored on day 0 of the experiment. Micofilariae production (B) was determined at each time point by counting the number of microfilaria present in 1 mL spent culture media. The data obtained from triplicate samples are expressed as a mean ± standard deviation.

Since filarial *Wolbachia* remain unculturable, we were unable to evaluate the direct effect of berberine on the endosymbiont. Therefore, we examined the indirect effect of the drug on adult female worm. As Figure 5A shows, berberine (10–40 µM) had adverse effects on the motility of adult female *B. malayi* worms, as well as microfilariae production (Figure 5B) when compared to untreated controls. Two days after treatment with berberine (40 µM), female worms showed almost no movement and the production of microfilaria had virtually ceased. Berberine at 20 µM was comparable to 10 µM of doxycycline in terms of effect on female worm motility. Reduction in adult female motility coincided with a decrease in microfilariae production. Similarly, motility of the freshly released microfilaria was decreased when berberine was present, with some effect observed at the lowest concentration (5 µM) tested (Figure 5C). On the other hand, male worms were more resistant to the effects of the drug with limited reduction in motility observed following treatment with berberine (5–40 µM) for 6 days (Figure 5D). However, treatment with 100 µM berberine for 24 h did completely paralyze male worms (data not shown). Doxycycline (10 µM) had a comparable affect on the motility of male and female worms.

### The effect of berberine on *E. coli* growth, morphology and viability

**Figure 6:**
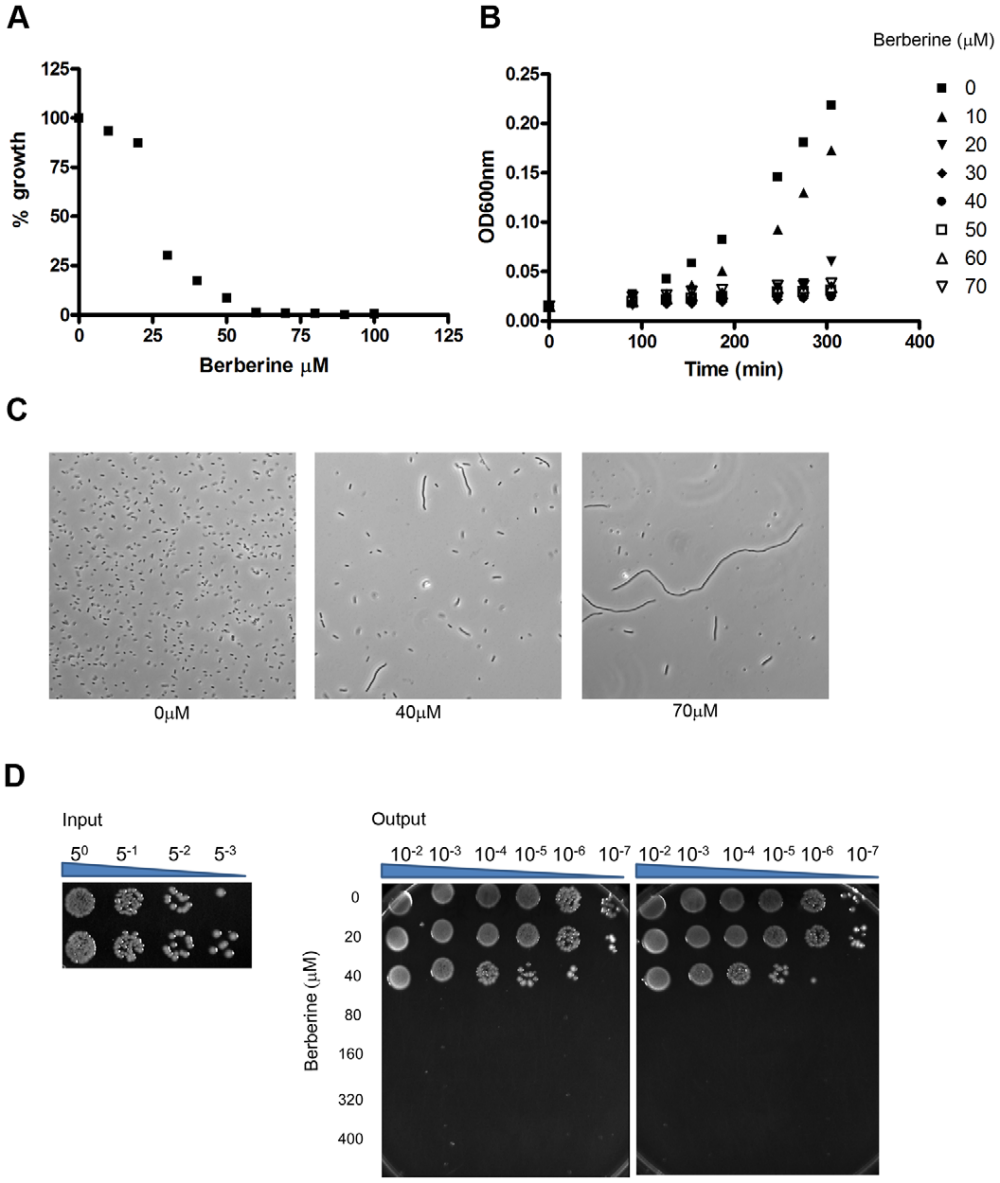
Berberine sulfate inhibition of *E. coli* growth. Panel A, overnight growth of *E. coli* was determined in the presence of various concentrations of berberine sulfate. Percentage of growth is indicated as 100×(OD_600_ nm with berberine/OD_600_ nm without berberine). The data obtained from triplicate samples are expressed as a mean ± standard deviation. Panel B, log-phase (5 h) growth (OD_600_ nm) of *E. coli* was determined in the presence of various concentrations (10–70 µM) of berberine sulphate. Panel C, DIC micrographs of *E. coli* untreated (0 µM) or treated with 40 µM or 80 µM berberine sulfate. Panel D, effect of berberine sulfate on *E. coli* viability. Viability of berberine sulfate-treated (24 hours) cells was evaluated by plating serial dilutions (10^−2^–10^−7^) of bacteria (Output shown in duplicate) on a petri dish and incubation overnight at 30 °C. The number of bacteria present in the inoculum used in the experiment (Input) is also shown.

To demonstrate that berberine's *in vitro* GTPase inhibitory activity and anti-parasitic activity correlates with its known antibacterial activity, studies were performed on *E. coli* strain ER1613. Berberine is known to act as a substrate for the multi-drug resistance efflux pumps and ER1613 contains a mutation in the *acrA* gene, which inactivates the multidrug efflux pump [45]. Overnight incubation of ER1613 with 0–100 µM berberine showed a dose-dependent effect with complete inhibition of bacterial growth observed at 60 µM (Figure 6A). Similarly, no growth was evident when experiments were initiated with greater bacterial densities and the cells were treated with 50 µM berberine for up to 5 h (Figure 6B). Treatment with berberine resulted in the filamentous phenotype (Figure 5C) typically observed in *ftsZ* mutant strains [46], indicating that berberine was inhibiting cell division. Moreover, the presence of elongated bacteria also correlated with decreased growth and viability. Viability was also evaluated by ability to form colonies on an agar plate. Berberine sulfate-treated (24 hours) cells produced substantially fewer colonies (Figure 6D), compared to untreated controls. Untreated bacteria had approximately 4×10^5^ - fold growth in 24 h, whereas bacteria treated with 40 µM berberine had 4×10^2^ - fold growth. At concentrations of 80 µM and higher, the treated bacteria failed to produce viable colonies (Figure 6D), demonstrating that without active replication *E. coli* die.

### Identification of new inhibitors of *w*Bm-FtsZ GTPase activity

**Figure 7:**
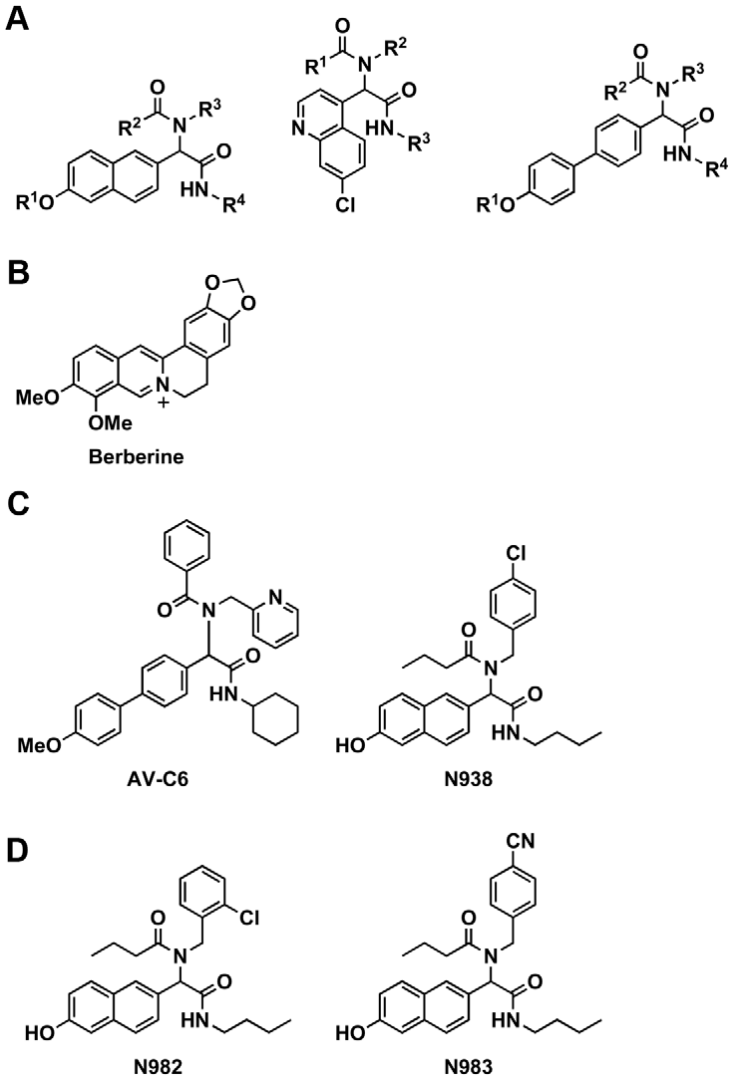
Structures of FtsZ inhibitors and scaffolds. General scaffolds for small molecule library compounds (A). Structure of berberine (B). FtsZ inhibitors identified from the initial high-throughput screen (C). FtsZ inhibitors identified from SAR studies (D).

**Figure 8:**
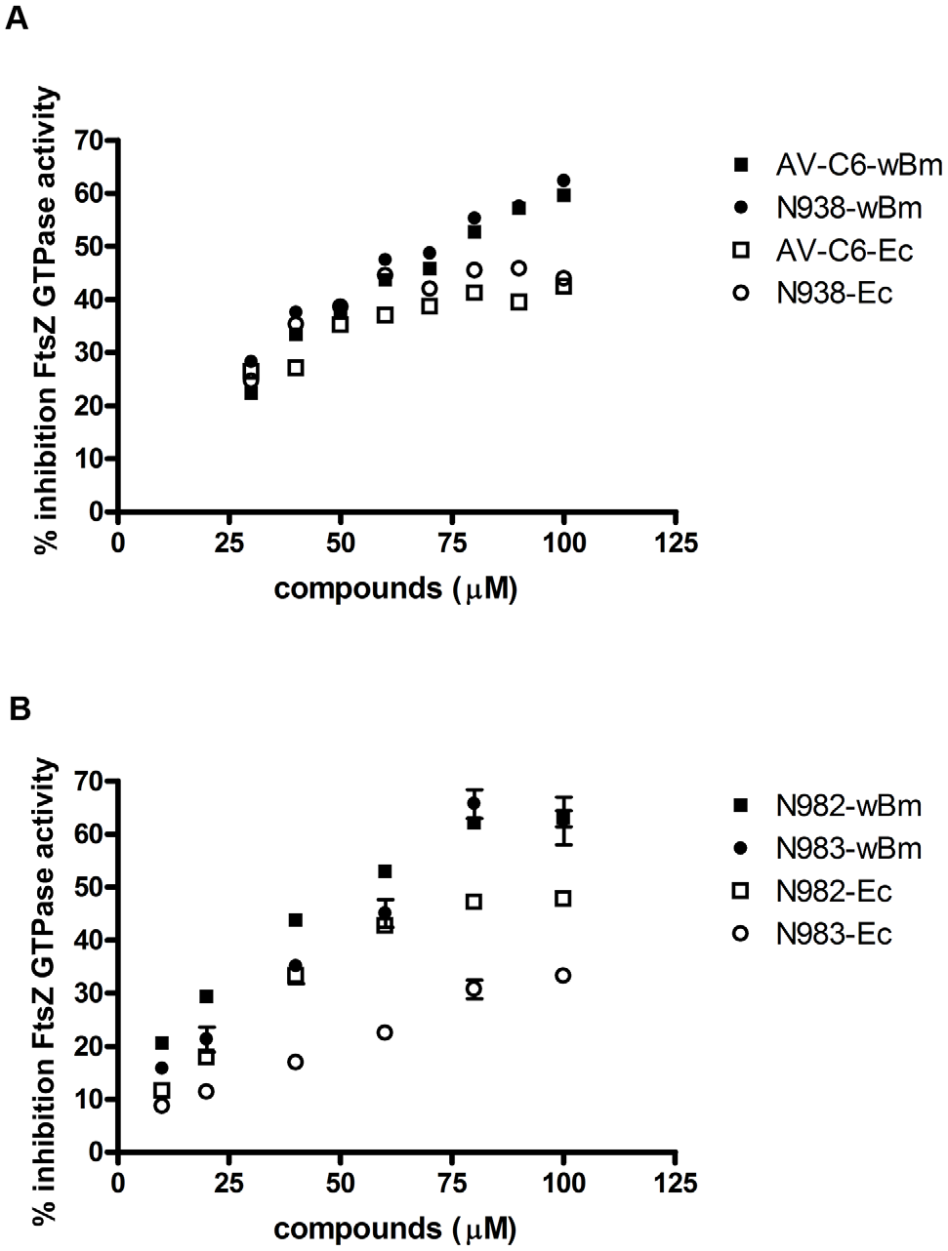
Inhibition of GTPase activity by small molecules. *w*Bm-FtsZ (▪ and •) and Ec-FtsZ (□ and ○) were compared. Panel A, compounds were tested at the concentration of 30, 40, 50, 60, 70, 80, 90, and 100 µM and the experiment were performed in duplicate, the mean value was plotted. Panel B, compounds were tested at the concentration of 10, 20, 40, 60, 80, and 100 µM and the experiments were performed in triplicate, the mean ± standard deviation was plotted.

To initiate a campaign to identify molecularly unique inhibitors of *w*Bm-FtsZ GTPase activity, a library of small molecules based on naphthalene, quinoline and biphenyl core scaffolds were examined [39]–[40] (Figure 7A). The library was constructed using Ugi multicomponent reaction chemistry, and each compound consists of a flat aromatic scaffold for enhanced π-stacking interactions decorated with varying diversity elements (R^1^–R^4^ in Figure 7A). Importantly, these scaffold motifs are also found in berberine (Figure 7B) and known FtsZ inhibitors [17], [29]–[33]. The ∼500-member library was screened using the *w*Bm-FtsZ GTPase assay, and 13 compounds with greater than 30% inhibition at 100 µM were identified. From these screening efforts, compounds AV-C6 and N938 (Figure 7C) emerged as leading hits, and each showed dose-dependent inhibition of *w*Bm-FtsZ (Figure 8A). AV-C6 and N938 were also examined for inhibition of the *E. coli* FtsZ enzyme (Figure 8A). As shown in Figure 8A, both compounds inhibited *Ec*-FtsZ activity although each was slightly less potent compared to the inhibitory activity against *w*Bm-FtsZ.

Structure-activity relationship (SAR) studies were then performed on N938 as this compound showed the most potential in dose response experiments. In addition to identifying compounds with enhanced potency, we were also interested in exploring the possibility of tuning down any inhibitory activity against *Ec*-FtsZ in order to obtain a more specific *Wolbachia* FtsZ inhibitor. A series of analogues were synthesized with varying aromatic side chains (R^3^ in Figure 7A). As shown in Figure 8B, both goals were met: N982 with an *ortho*-chloro substituent (Figure 7D) showed enhanced potency in the *w*Bm-FtsZ assay and N983 with a *para*-cyano substituent (Figure 7D) showed some specificity for *w*Bm-FtsZ over that from *E. coli*. Future SAR studies should enable the discovery of compounds with both enhanced inhibitory properties and specificity. Finally, as the solubility of these compounds is poor, 100% inhibition of FtsZ with this scaffold was not possible and true IC_50_ values could not be obtained. Scaffold modification and/or hopping strategies will be investigated in the future to afford enhanced solubility.

## Discussion

The use of antibiotics targeting the *Wolbachia* endosymbionts of filarial parasites has been validated as an approach for controlling filarial infection in animals and humans. As a result, there is considerable interest in identifying new compounds that specifically target the obligate bacterial endosymbiont. In the present study, we investigated the cell division pathway in *w*Bm to identify new drug targets that may be exploited for the development of new antifilarial therapies. Filamenting temperature sensitive (*fts*) genes produce many of the proteins essential for cell division in *E. coli* [17]. In *w*Bm, we identified the majority of core genes that are indispensable to cytokinesis including *ftsA*, *ftsI*, *ftsK*, *ftsQ*, *ftsW* and *ftsZ*.

Interestingly, *ftsB*, *ftsL*, *ftsN* and *ZipA* were not found in *w*Bm. ZipA is a bitopic membrane protein with a large cytoplasmic domain that binds and bundles FtsZ protofilaments *in vitro* and helps to stabilize the Z ring *in vivo*. FtsN is a core component of the divisome that accumulates at the septal ring at the initiation of the constriction process. The C-terminal SPOR domain specifically recognizes a transient form of septal murein, which helps trigger and sustain the constriction process. However, in *E. coli*, it has been found that alterations in FtsA can compensate for the absence of ZipA, FtsK [47] and FtsN [48] and a gain-of-function FtsA variant, FtsA*(R286W), efficiently stimulates cell division in the complete absence of ZipA [47]. Thus, *Wolbachia* FtsA may function like the mutant FtsA, as an alanine residue is present in the same position.

*ftsB*, *ftsL*, *ftsN* and *ZipA* are also absent in some important bacterial pathogens including certain Gram-negative (*Neisseria spp.*, *Bordetella pertussis*, *Helicobacter pylori*, *Chlamydia spp.*) and Gram-positive (*Mycobacterium tuberculosis*) bacteria and cell wall-lacking (*Mycoplasma pneumoniae*) organisms [17]. It is likely that this reflects the reduced genome size present in these intracellular bacteria.

FtsZ is the most highly conserved essential bacterial cell division protein and is present in all bacteria except *Chlamydia spp* [17]. We determined that *w*Bm-FtsZ shares substantial similarity (43% identity) to the highly characterized *E. coli* FtsZ protein and is highly similar (∼90% identity) to insect *Wolbachia* FtsZ proteins. While the majority of *w*Bm genes are expressed in a stage-specific manner [49], *w*Bm-*fts*Z was found to be expressed in both male and female worms as well as in all larval stages examined. It was not surprising to find *w*Bm-*ftsZ* expressed throughout the entire lifecycle of the parasite since the bacterial Z-ring is known to exist in a state of dynamic equilibrium in order to fulfill its many roles in the cell. Using fluorescence recovery after photo bleaching (FRAP), the *E. coli* Z-ring was found to continually remodel itself with a halftime of 30 seconds with only 30% of cellular FtsZ present in the ring with continuous and rapid exchange of subunits within a cytoplasmic pool [17]. *E. coli ftsZ* transcription analysis has revealed that the rate of *ftsZ* expression is constant with a sudden doubling at a specific cell age, suggesting that *ftsZ* expression is regulated [50]. Similarly, we observed up-regulation of *w*Bm-*ftsZ* gene expression in fourth-stage larvae and adult female worms with microfilariae likely contributing to the increased expression in the latter case. While the lowest levels of gene expression were evident in adult males, FtsZ protein was easily detected in proteomic analyses of male worms [49]. In general, the gene expression pattern of *ftsZ* correlated with bacterial multiplication. The increased bacterial multiplication in the worm during early infection of the mammalian host and embryogenesis is in agreement with an earlier study [4]. These data are consistent with the third- and fourth-stage larval stages, and embryogenesis being particularly sensitive to the effects of antibiotic treatment [4], [51]. This result indicates that *ftsZ* gene expression could be used as a marker to monitor *Wolbachia* multiplication in the filarial parasite much like the *ftsZ* gene in the intracellular bacterium *Candidatus Glomeribacter gigasporarum* that resides in the mycorrhizal fungus *Gigaspora margarita* [43].

Molecular studies have established the importance of conserved amino acids in the FtsZ protein that when changed results in *ftsZ* mutants blocked at different stages of cell division [42], [46], [52]–[55]. *w*Bm-FtsZ possesses the key residues and conserved GTP-binding pocket required for GTPase activity. Our functional analysis revealed that the GTPase activities of recombinant *w*Bm-FtsZ and Ec-FtsZ are similar, and both proteins are sensitive to the plant alkaloid berberine. Most of the residues in Ec-FtsZ that are thought to bind berberine and inhibit FtsZ GTPase activity are also present in *w*Bm-FtsZ. An earlier detailed study in *E. coli* determined that the target of this commonly used compound is FtsZ [33]. Plants containing berberine have been used in traditional Chinese and Native American medicine to treat many infectious diseases and the sulfate, hydrochloride and chloride forms are used in Western pharmaceutical medicine as antibacterial agents [56]. It is active against a number of Gram-positive and Gram-negative pathogenic bacteria, including drug resistant *Mycobacterium tuberculosis* [57] and *Staphylococcus aureus* [58].

Our experiments in *E. coli* demonstrate that berberine has both bacteriostatic and bacteriocidal effects. Since filarial *Wolbachia* remain unculturable, we were unable to evaluate the direct effect of berberine on the endosymbiont. However, following berberine treatment, we did observe reductions in adult female worm and microfilariae motility and microfilariae production. On the other hand, we did not see any effect on male worms, which had the lowest level of *w*Bm-*ftsZ* gene expression. We examined berberine- and doxycycline-treated worms for *Wolbachia* load by qPCR analysis and did not observe a significant difference between control and treated parasites. A similar result was also found in a study evaluating the effects of globomycin and doxycycline on filarial *Wolbachia*, and the authors [59] suggested several possibilities which can also apply to our study, namely: the *Wolbachia* qPCR assay may not have sufficient sensitivity to detect effects on *Wolbachia* load over this time frame in nematodes, inhibition of FtsZ is sufficient to affect nematode motility and viability independent of or prior to any effect on *Wolbachia* load, and/or a direct effect of berberine on nematode motility and viability and alternative mechanisms of action. Nonetheless, our results suggest that FtsZ inhibitors that operate via inhibition of enzyme activity including natural products [28], [30]–[33], [53] and synthetic molecules [29], [60] may have also activity against *w*Bm-FtsZ.

To complement the berberine studies, a library of naphthalene-, quinoline- and biphenyl-based compounds constructed using Ugi multicomponent reaction chemistry was examined for the discovery of new and ultimately highly specific antagonists of either *E. coli* or *Wolbachia* FtsZ. Of interest, compounds based on similar scaffolds have already been demonstrated as potent FtsZ inhibitors [17], [29]–[33]. From our screening efforts, the (6-{butylcarbamoyl-[(aryl)-(butylcarbonyl)-amino]-methyl})-naphthen-2-ol scaffold (Figure 7A, C) emerged as an antagonist of both *E. coli* and *Wolbachia* FtsZ. Interestingly, from basic SAR studies it appears that modification of the aryl substituent on the scaffold may afford selectivity for *Wolbachia* FtsZ, a key element of our initial goal. Additional compounds are currently being prepared to examine this possibility. Although not discussed here, compounds based on our lead scaffold had no effect on growth or viability in *E. coli*. Based on these findings and their potency in the *in vitro* assays, it is plausible that penetrability or metabolism issues are to blame for their attenuated activity. Finally, the solubility of these compounds is also poor precluding measurement of true IC_50_ values. Further iterations of chemical synthesis will be necessary to address these potential liabilities.

While we have focused on assaying the GTPase activity of *w*Bm-FtsZ using a medium- to high-throughput coupled enzyme assay for the discovery of inhibitors that target cell division in *Wolbachia*, it is also possible to screen for compounds that would target *w*Bm-FtsZ via other mechanisms of action. FtsZ is considered a distant functional relative of the mammalian cytoskeletal protein β-tubulin [61]–[63]. Microtubule formation is a major target in cancer chemotherapy and the anticancer drug Taxol binds to β-tubulin and blocks cell division by interfering with microtubule formation. Interestingly, the FtsZ inhibitor PC190723 [60] operates by a similar mechanism and more recently, novel inhibitors of *B. subtilis* cell division have been identified in an *in vitro* FtsZ protofilaments polymerization assay [64]. Importantly, significant differences exist in the active sites in tubulin and FtsZ polymers, and several small molecule inhibitors of FtsZ have been identified [65] that do not inhibit tubulin [66]–[67]. Tubulin is also the target of the broadly anti-parasitic benzimidazole drugs [68]–[69], which have been used extensively to control soil-transmitted nematodes [70]–[71].

FtsZ is also responsible for recruiting and coordinating more than a dozen other cell division proteins at the midcell site of the closing septum [18]–[19], [21], [72]. Many of these interactions are essential and it has been suggested that they might also be useful targets, particularly in light of developments in the discovery of small molecule inhibitors of protein-protein interactions [17], [73]–[74]. Therefore, it might be feasible to screen for inhibitors of the interactions between *w*Bm-FtsZ and its various binding partners that modulate its polymerization. Another *Wolbachia* cell division protein worth considering for drug discovery is FtsA, as this protein also possesses enzymatic activity and contains an ATP-binding site that might be targeted with drug-like molecules. Moreover, this protein is essential in *E. coli* [75] and *Streptococcus pneumoniae* [76].

In summary, we have investigated the cell division pathway in *w*Bm and determined that it possesses a FtsZ protein with GTPase activity. We demonstrated that the activity is inhibited by berberine and identified small molecule inhibitors in a high-throughput screen. Furthermore, berberine was found to have adverse affects on *B. malayi* adult worm and microfilariae motility, and reproduction. Our results support the discovery of selective inhibitors of *Wolbachia* FtsZ as a new therapeutic approach for filariasis.

## References

[1] Taylor MJ, Bandi C, Hoerauf A (2005). Wolbachia bacterial endosymbionts of filarial nematodes.. Adv Parasitol.

[2] Kozek WJ (1977). Transovarially-transmitted intracellular microorganisms in adult and larval stages of Brugia malayi.. J Parasitol.

[3] Taylor MJ, Bilo K, Cross HF, Archer JP, Underwood AP (1999). 16S rDNA phylogeny and ultrastructural characterization of Wolbachia intracellular bacteria of the filarial nematodes Brugia malayi, B. pahangi, and Wuchereria bancrofti.. Exp Parasitol.

[4] McGarry HF, Egerton GL, Taylor MJ (2004). Population dynamics of Wolbachia bacterial endosymbionts in Brugia malayi.. Mol Biochem Parasitol.

[5] Bandi C, McCall JW, Genchi C, Corona S, Venco L (1999). Effects of tetracycline on the filarial worms Brugia pahangi and Dirofilaria immitis and their bacterial endosymbionts Wolbachia.. Int J Parasitol.

[6] Hoerauf A, Specht S, Marfo-Debrekyei Y, Buttner M, Debrah AY (2009). Efficacy of 5-week doxycycline treatment on adult Onchocerca volvulus.. Parasitol Res.

[7] Turner JD, Tendongfor N, Esum M, Johnston KL, Langley RS (2010). Macrofilaricidal activity after doxycycline only treatment of Onchocerca volvulus in an area of Loa loa co-endemicity: a randomized controlled trial.. PLoS Negl Trop Dis.

[8] Mand S, Pfarr K, Sahoo PK, Satapathy AK, Specht S (2009). Macrofilaricidal activity and amelioration of lymphatic pathology in bancroftian filariasis after 3 weeks of doxycycline followed by single-dose diethylcarbamazine.. Am J Trop Med Hyg.

[9] Hoerauf A (2006). New strategies to combat filariasis.. Expert Rev Anti Infect Ther.

[10] Taylor MJ, Makunde WH, McGarry HF, Turner JD, Mand S (2005). Macrofilaricidal activity after doxycycline treatment of Wuchereria bancrofti: a double-blind, randomised placebo-controlled trial.. Lancet.

[11] Foster J, Ganatra M, Kamal I, Ware J, Makarova K (2005). The Wolbachia genome of Brugia malayi: endosymbiont evolution within a human pathogenic nematode.. PLoS Biol.

[12] Kumar S, Chaudhary K, Foster JM, Novelli JF, Zhang Y (2007). Mining Predicted Essential Genes of Brugia malayi for Nematode Drug Targets.. PLoS ONE.

[13] Holman AG, Davis PJ, Foster JM, Carlow CK, Kumar S (2009). Computational prediction of essential genes in an unculturable endosymbiotic bacterium, Wolbachia of Brugia malayi.. BMC Microbiol.

[14] Foster JM, Zhang Y, Kumar S, Carlow CK (2005). Mining nematode genome data for novel drug targets.. Trends Parasitol.

[15] Carballido-Lopez R, Errington J (2003). A dynamic bacterial cytoskeleton.. Trends Cell Biol.

[16] Graumann PL (2007). Cytoskeletal elements in bacteria.. Annu Rev Microbiol.

[17] Lock RL, Harry EJ (2008). Cell-division inhibitors: new insights for future antibiotics.. Nat Rev Drug Discov.

[18] Bi EF, Lutkenhaus J (1991). FtsZ ring structure associated with division in Escherichia coli.. Nature.

[19] Margolin W (2005). FtsZ and the division of prokaryotic cells and organelles.. Nat Rev Mol Cell Biol.

[20] Arends SJ, Weiss DS (2004). Inhibiting cell division in Escherichia coli has little if any effect on gene expression.. J Bacteriol.

[21] Vollmer W (2008). Targeting the bacterial Z-ring.. Chem Biol.

[22] Rastogi N, Domadia P, Shetty S, Dasgupta D (2008). Screening of natural phenolic compounds for potential to inhibit bacterial cell division protein FtsZ.. Indian J Exp Biol.

[23] Dasgupta D (2009). Novel compound with potential of an antibacterial drug targets FtsZ protein.. Biochem J.

[24] Slayden RA, Knudson DL, Belisle JT (2006). Identification of cell cycle regulators in Mycobacterium tuberculosis by inhibition of septum formation and global transcriptional analysis.. Microbiology.

[25] Huang Q, Kirikae F, Kirikae T, Pepe A, Amin A (2006). Targeting FtsZ for antituberculosis drug discovery: noncytotoxic taxanes as novel antituberculosis agents.. J Med Chem.

[26] Addinall SG, Small E, Whitaker D, Sturrock S, Donachie WD (2005). New temperature-sensitive alleles of ftsZ in Escherichia coli.. J Bacteriol.

[27] Margolin W, Bernander R (2004). How do prokaryotic cells cycle?. Curr Biol.

[28] Andreu JM, Schaffner-Barbero C, Huecas S, Alonso D, Lopez-Rodriguez ML (2010). The antibacterial cell division inhibitor PC190723 is an FtsZ polymer-stabilizing agent that induces filament assembly and condensation.. J Biol Chem.

[29] Wang J, Galgoci A, Kodali S, Herath KB, Jayasuriya H (2003). Discovery of a small molecule that inhibits cell division by blocking FtsZ, a novel therapeutic target of antibiotics.. J Biol Chem.

[30] Beuria TK, Santra MK, Panda D (2005). Sanguinarine blocks cytokinesis in bacteria by inhibiting FtsZ assembly and bundling.. Biochemistry.

[31] Domadia P, Swarup S, Bhunia A, Sivaraman J, Dasgupta D (2007). Inhibition of bacterial cell division protein FtsZ by cinnamaldehyde.. Biochem Pharmacol.

[32] Jaiswal R, Beuria TK, Mohan R, Mahajan SK, Panda D (2007). Totarol inhibits bacterial cytokinesis by perturbing the assembly dynamics of FtsZ.. Biochemistry.

[33] Domadia PN, Bhunia A, Sivaraman J, Swarup S, Dasgupta D (2008). Berberine targets assembly of Escherichia coli cell division protein FtsZ.. Biochemistry.

[34] O'Neill SL, Pettigrew MM, Sinkins SP, Braig HR, Andreadis TG (1997). In vitro cultivation of Wolbachia pipientis in an Aedes albopictus cell line.. Insect Mol Biol.

[35] Baldo L, Dunning Hotopp JC, Jolley KA, Bordenstein SR, Biber SA (2006). Multilocus sequence typing system for the endosymbiont Wolbachia pipientis.. Appl Environ Microbiol.

[36] Margalit DN, Romberg L, Mets RB, Hebert AM, Mitchison TJ (2004). Targeting cell division: small-molecule inhibitors of FtsZ GTPase perturb cytokinetic ring assembly and induce bacterial lethality.. Proc Natl Acad Sci U S A.

[37] Rao RU, Moussa H, Weil GJ (2002). Brugia malayi: effects of antibacterial agents on larval viability and development in vitro.. Exp Parasitol.

[38] Rao R, Well GJ (2002). In vitro effects of antibiotics on Brugia malayi worm survival and reproduction.. J Parasitol.

[39] Xu Y, Shi J, Yamamoto N, Moss JA, Vogt PK (2006). A credit-card library approach for disrupting protein-protein interactions.. Bioorg Med Chem.

[40] Xu Y, Lu H, Kennedy JP, Yan X, McAllister LA (2006). Evaluation of “credit card” libraries for inhibition of HIV-1 gp41 fusogenic core formation.. J Comb Chem.

[41] Scheffers DJ, de Wit JG, den Blaauwen T, Driessen AJ (2002). GTP hydrolysis of cell division protein FtsZ: evidence that the active site is formed by the association of monomers.. Biochemistry.

[42] Lu C, Stricker J, Erickson HP (2001). Site-specific mutations of FtsZ–effects on GTPase and in vitro assembly.. BMC Microbiol.

[43] Anca IA, Lumini E, Ghignone S, Salvioli A, Bianciotto V (2009). The ftsZ gene of the endocellular bacterium ‘Candidatus Glomeribacter gigasporarum’ is preferentially expressed during the symbiotic phases of its host mycorrhizal fungus.. Mol Plant Microbe Interact.

[44] Boberek JM, Stach J, Good L (2010). Genetic evidence for inhibition of bacterial division protein FtsZ by berberine.. PLoS One.

[45] Tegos G, Stermitz FR, Lomovskaya O, Lewis K (2002). Multidrug pump inhibitors uncover remarkable activity of plant antimicrobials.. Antimicrob Agents Chemother.

[46] Bi E, Lutkenhaus J (1992). Isolation and characterization of ftsZ alleles that affect septal morphology.. J Bacteriol.

[47] Geissler B, Shiomi D, Margolin W (2007). The ftsA* gain-of-function allele of Escherichia coli and its effects on the stability and dynamics of the Z ring.. Microbiology.

[48] Dai K, Xu Y, Lutkenhaus J (1993). Cloning and characterization of ftsN, an essential cell division gene in Escherichia coli isolated as a multicopy suppressor of ftsA12(Ts).. J Bacteriol.

[49] Bennuru S, Meng Z, Ribeiro JM, Semnani RT, Ghedin E (2011). Stage-specific proteomic expression patterns of the human filarial parasite Brugia malayi and its endosymbiont Wolbachia.. Proc Natl Acad Sci U S A.

[50] Robin A, Joseleau-Petit D, D'Ari R (1990). Transcription of the ftsZ gene and cell division in Escherichia coli.. J Bacteriol.

[51] Hoerauf A, Nissen-Pahle K, Schmetz C, Henkle-Duhrsen K, Blaxter ML (1999). Tetracycline therapy targets intracellular bacteria in the filarial nematode Litomosoides sigmodontis and results in filarial infertility.. J Clin Invest.

[52] Addinall SG, Bi E, Lutkenhaus J (1996). FtsZ ring formation in fts mutants.. J Bacteriol.

[53] Stricker J, Erickson HP (2003). In vivo characterization of Escherichia coli ftsZ mutants: effects on Z-ring structure and function.. J Bacteriol.

[54] Wang Y, Jones BD, Brun YV (2001). A set of ftsZ mutants blocked at different stages of cell division in Caulobacter.. Mol Microbiol.

[55] Redick SD, Stricker J, Briscoe G, Erickson HP (2005). Mutants of FtsZ targeting the protofilament interface: effects on cell division and GTPase activity.. J Bacteriol.

[56] Kong DX, Li XJ, Tang GY, Zhang HY (2008). How many traditional Chinese medicine components have been recognized by modern Western medicine? A chemoinformatic analysis and implications for finding multicomponent drugs.. ChemMedChem.

[57] Gentry EJ, Jampani HB, Keshavarz-Shokri A, Morton MD, Velde DV (1998). Antitubercular natural products: berberine from the roots of commercial Hydrastis canadensis powder. Isolation of inactive 8-oxotetrahydrothalifendine, canadine, beta-hydrastine, and two new quinic acid esters, hycandinic acid esters-1 and -2.. J Nat Prod.

[58] Yu HH, Kim KJ, Cha JD, Kim HK, Lee YE (2005). Antimicrobial activity of berberine alone and in combination with ampicillin or oxacillin against methicillin-resistant Staphylococcus aureus.. J Med Food.

[59] Johnston KL, Wu B, Guimaraes A, Ford L, Slatko BE (2010). Lipoprotein biosynthesis as a target for anti-Wolbachia treatment of filarial nematodes.. Parasit Vectors.

[60] Haydon DJ, Stokes NR, Ure R, Galbraith G, Bennett JM (2008). An inhibitor of FtsZ with potent and selective anti-staphylococcal activity.. Science.

[61] Lowe J, Amos LA (1998). Crystal structure of the bacterial cell-division protein FtsZ.. Nature.

[62] Nogales E, Wolf SG, Downing KH (1998). Structure of the alpha beta tubulin dimer by electron crystallography.. Nature.

[63] Downing KH (2000). Structural basis for the interaction of tubulin with proteins and drugs that affect microtubule dynamics.. Annu Rev Cell Dev Biol.

[64] Beuria TK, Singh P, Surolia A, Panda D (2009). Promoting assembly and bundling of FtsZ as a strategy to inhibit bacterial cell division: a new approach for developing novel antibacterial drugs.. Biochem J.

[65] Vollmer W (2006). The prokaryotic cytoskeleton: a putative target for inhibitors and antibiotics?. Appl Microbiol Biotechnol.

[66] Lappchen T, Hartog AF, Pinas VA, Koomen GJ, den Blaauwen T (2005). GTP analogue inhibits polymerization and GTPase activity of the bacterial protein FtsZ without affecting its eukaryotic homologue tubulin.. Biochemistry.

[67] Lappchen T, Pinas VA, Hartog AF, Koomen GJ, Schaffner-Barbero C (2008). Probing FtsZ and tubulin with C8-substituted GTP analogs reveals differences in their nucleotide binding sites.. Chem Biol.

[68] Lubega GW, Prichard RK (1991). Specific interaction of benzimidazole anthelmintics with tubulin from developing stages of thiabendazole-susceptible and -resistant Haemonchus contortus.. Biochem Pharmacol.

[69] Lacey E (1988). The role of the cytoskeletal protein, tubulin, in the mode of action and mechanism of drug resistance to benzimidazoles.. Int J Parasitol.

[70] McCracken RO, Lipkowitz KB (1990). Structure-activity relationships of benzothiazole and benzimidazole anthelmintics: a molecular modeling approach to in vivo drug efficacy.. J Parasitol.

[71] Prichard RK (2007). Markers for benzimidazole resistance in human parasitic nematodes?. Parasitology.

[72] Weiss DS (2004). Bacterial cell division and the septal ring.. Mol Microbiol.

[73] Tsao DH, Sutherland AG, Jennings LD, Li Y, Rush TS (2006). Discovery of novel inhibitors of the ZipA/FtsZ complex by NMR fragment screening coupled with structure-based design.. Bioorg Med Chem.

[74] Jennings LD, Foreman KW, Rush TS, Tsao DH, Mosyak L (2004). Design and synthesis of indolo[2,3-a]quinolizin-7-one inhibitors of the ZipA-FtsZ interaction.. Bioorg Med Chem Lett.

[75] Donachie WD, Begg KJ, Lutkenhaus JF, Salmond GP, Martinez-Salas E (1979). Role of the ftsA gene product in control of Escherichia coli cell division.. J Bacteriol.

[76] Lara B, Rico AI, Petruzzelli S, Santona A, Dumas J (2005). Cell division in cocci: localization and properties of the Streptococcus pneumoniae FtsA protein.. Mol Microbiol.

